# Cross-language validation of COVID-19 Compliance Scale in 28 languages

**DOI:** 10.1017/S0950268823001103

**Published:** 2023-07-10

**Authors:** Angélique M. Blackburn, Hyemin Han, Aranza Gallegos

**Affiliations:** 1 Department of Psychology and Communication, Texas A&M International University, Laredo, TX, USA; 2 Educational Psychology Program, University of Alabama, Tuscaloosa, AL, USA

**Keywords:** compliance, COVID-19, cross-language survey, public health, survey validation

## Abstract

Although compliance scales have been used to assess compliance with health guidelines to reduce the spread of COVID-19, no scale known to us has shown content validity regarding global guidelines and reliability across an international sample. We assessed the validity and reliability of a Compliance Scale developed by a group of over 150 international researchers. Exploratory factor analysis determined reliable items on the English version. Confirmatory factor analysis confirmed the reliability of the six-item scale and convergent validity was found. After invariance testing and alignment, we employed a novel *R* code to run a Monte Carlo simulation for alignment validation. This scale can be employed to measure compliance across multiple languages, and our alignment validation method can be conducted with future cross-language surveys.

## Introduction

Increasing individuals’ compliance with health guidelines is critical to reducing the spread of disease, especially in the case of highly contagious diseases like the COVID-19 pandemic. Early in the COVID-19 pandemic, ways to reduce its spread were adapted from knowledge of other epidemics and were tested quickly for efficacy. These measures included wearing a face mask, washing hands, avoiding crowded areas, staying home, avoiding face-to-face social interactions, self-isolating while symptomatic or sick, and spatially social distancing [1, 2].

Convincing the public to comply with local health guidelines has proved difficult in the past [3], and this was also the case with the global COVID-19 pandemic [1, 4]. Barriers to compliance in some contexts have been identified, such as difficulty maintaining social distancing in public situations, low risk perception, social pressures, difficulties with changing rules, and mental health reasons including the need for social support and reducing social isolation [4]. Compliance with guidelines has differed across countries and demographics. For instance, compliance tends to be higher in women than men [5–8], older adults than younger adults [5, 6] (c.f., [7]), some racial minority groups [9], and groups with lower SES and education levels [5]. In addition, personality traits [10, 11], political affiliation [12], fear of COVID-19 [13], moral values [13, 14], and risk aversion [12] have all been associated with compliance.

To understand the most effective methods to ensure compliance, it is important to understand the factors that contribute to increased compliance. Compliance scales have been used to measure compliance with COVID-19 guidelines across countries, demographics, and time (e.g., at the beginning of the pandemic and a year into the COVID-19 pandemic; [15, 16]).

### Existing compliance scales

In order to compare compliance measures and efficacy across countries, demographics, and time, a universal scale must be developed and tested. There are currently a number of existing scales to measure compliance with health guidelines, but none of them show strong content validity for compliance specifically as well as having been validated in a global sample across multiple languages and dialects.

In Supplementary Table S1, we have presented the most prevalent compliance scales. The majority of these scales have been used in only one country. A number of compliance scales have been administered in previous studies in only one language, often English, but some of these scales were developed and/or translated into other languages (see Supplementary Table S1 for more details regarding languages and countries). These studies showed reliable results within specific countries and languages but were not administered or validated in a global setting.

A few compliance scales have been translated and administered globally in multiple languages. Van Bavel et al. [17] sampled from 67 countries and territories. However, their scale was not one comprehensive scale solely about compliance, but rather included three sub-scales that measured compliance-related items such as the Physical Hygiene Scale, the Spatial Distancing Scale, and the Policy Support scale. Each of these scales had at least moderate reliability (**α** > 0.60), with the exception of the full spatial distance scale, which had lower reliability. This is a strong scale that has been implemented in follow-up studies regarding COVID-19 compliance. For instance, Lin et al. [5] administered this scale across countries and found that older adults, women, and individuals in countries with a low human development index (HDI) reported greater compliance[5]. In countries with higher HDI, gender differences in compliance were observed, with women reporting greater levels of compliance.

Plohl and Musil [18] also administered a strong global scale, but it was slightly longer (11 items), and social distancing was addressed in three items[18]. Thus, there are global scales for compliance that were validated across countries, but these scales each focused separately on spatial distance/physical contact, hygiene, and policy support [17] or focused more on certain aspects of compliance than others [18]. In contrast, a short, rapid scale with high content validity and reliability that measures multiple aspects of compliance is needed.

We also created an early version of a compliance scale as part of an initial COVIDiSTRESS Global Survey in 2020 [15]. This scale was administered in 176 countries, with 42 having a large enough sample size to be included in survey validation. This scale was created in English and subsequently translated into 48 languages and dialects. However, our initial scale was not found to represent one underlying latent factor, so individual compliance items were used in follow-up analyses rather than using the full scale [10, 19]. Our initial compliance scale contained six items, but we determined that a stronger scale would have items that related more closely to public health guideline compliance.

To measure compliance and effectively compare studies across different cultures and languages, it is necessary to have a global scale of compliance related to COVID-19 with high content validity and reliability. We created such a scale as part of the COVIDiSTRESS II Global Survey. Importantly, this scale was created with input from roughly 150 international Consortium members from over 50 countries to ensure that the measures represented global guidelines that were consistently promoted across many countries. Many of these researchers have experience designing and validating multilingual surveys across languages (e.g., [20–23]). The scale was first written in English, then translated into 48 languages using a three-step translation, back-translation, and verification process [16].

### This study

In this study, we tested the reliability and validity of the Compliance Scale from the COVIDiSTRESS II Global Survey, which was administered online in 137 countries and 48 languages and dialects during the summer of 2021 [16]. We tested the validity of the Compliance Scale using exploratory factor analysis (EFA), confirmatory factor analysis (CFA), measurement invariance testing and alignment, convergence with vaccine willingness, and known-groups validity testing using age and gender comparisons.

We tested whether after items had been removed via EFA, the remaining items on the scale reflected the same number of constructs during CFA, each with acceptable internal consistency. We predicted that after the removal of pre-determined items based on theory, the scale would reflect one underlying latent variable. We then conducted a measurement invariance test to determine whether the different language versions of the scale are measuring the same construct with the same measurement structure [24]. Invariance or measurement alignment in the case of invariance is critical to ensure that the scales are comparable across languages and cultures [20, 25]. In the event of measurement non-variance, measurement alignment was also planned. We then assessed the convergent validity of the scale; we predicted that COVID-19 compliance, as measured by the shortened scale, would correlate with vaccine willingness, another form of compliance to reduce the spread of disease. Finally, we conducted two known-groups comparisons based on age and gender. Both age and gender have robust, replicated associations with compliance. Higher compliance is usually observed in elderly adults than young adults, and higher compliance is observed in women compared to men (e.g., [6, 7]). Therefore, we predicted that elderly individuals and women would have greater compliance than young adults and men, respectively.

## Methods

### Transparency and openness

The analysed dataset is available in the Open Science Framework repository: (https://osf.io/36tsd/). Data collection occurred online as described in Blackburn et al.[16] and was pre-registered (https://osf.io/pg3h8). The analyses described herein were pre-registered after data collection, yet before data analysis (https://osf.io/xt4ru). Ethics approval was granted by the University of Salford (ref. 1632).

Statistical models were conducted in R (EFA was verified in SPSS, with agreement between methods; SPSS not reported herein), using methods described elsewhere [20, 25]. Analyses were conducted with customised R codes [26] and codes are available at: https://github.com/Neuropinklab/Identity-Project; https://github.com/Neuropinklab/Compliance_Validation.git.

### Population

After data cleaning, 15,740 participants were included in the dataset. Only countries with sample sizes greater than 100 per language were included to allow for measurement invariance analyses. This resulted in a sample set of 15,103 participants across 115 countries.

### Scales

We analysed the following variables as measured by the COVIDiSTRESS II Global Survey: vaccine willingness and compliance as measured using the Compliance Scale (CS) adapted from scales presented in Supplementary Table S1. The Compliance Scale asked participants to indicate their agreement (1 = strongly disagree; 7 = strongly agree) with whether they exhibited specific behaviors over the past month (e.g., washed hands regularly).Vaccine willingness was measured as a single item about willingness to receive the COVID-19 vaccine if it were available, with an additional Vaccine Attitude Scale measured separately as described elsewhere [25].

#### EFA

We tested the validity and reliability of the Compliance Scale in the English survey, as the survey was originally created in English, using a randomly selected half of the English language participants (N = 795). For the EFA analysis, we imported *EFAtools* and *psych* [27, 28]. EFA was conducted independently by two authors, in R and SPSS, respectively, to determine agreement. Both authors independently arrived at the same shortened survey solution, so only the R results are reported here. During the EFA, items were removed based on theory, and we determined if internal consistency and loadings improved when they were removed. In particular, items 7 and then 3 were pre-determined to be excluded stepwise because COVIDiSTRESS Consortium members considered that these items may be based on local regulations and participants’ interpretation of the questions.

#### CFA

The CFA was based on EFA results and conducted with the data of the remaining English language participants (N = 795). For this analysis, we imported *lavaan* [29] and *psych* [27]. We employed the WLSMV estimator because response options were on a Likert scale. This uses diagonally weighted least squares (DWLS) to estimate the model parameters. It uses the full weight matrix to compute mean/variance-adjusted values and robust standard errors. For acceptable internal consistency of each factor on the scale, Cronbach’s alpha level greater than 0.6 was required. Factor loadings above 0.4 were considered appropriate. If the requirements for EFA and CFA indicators were not fulfilled, we planned to continue adjusting the items and re-testing the revised scale.

#### Validity testing

Once we determined how many latent variables were represented on the Compliance Scale and the items with the best factor loadings, we 1) tested invariances and alignment across languages 2) tested convergent validity by analysing the relationship between compliance (six items) and vaccine willingness (1 item), 3) tested known-groups validity using gender by comparing men and women, and 4) tested known-groups validity using age by comparing young adults aged 18–39 to elderly adults aged 60+. Additionally, to include all ages in the validation, a correlation between age and compliance was conducted. For the known-groups validity tests, we also conducted Bayesian tests to examine whether data directly supported our alternative hypotheses instead of the null hypotheses [30]. When Bayes Factors (BF), which resulted from the conducted tests, were three or higher, we concluded that evidence positively supported the presence of a non-zero effect (e.g., significant difference or correlation) [31].

#### Invariance testing

For invariance testing, we imported packages *lavaan* [29] for CFA, *sirt* [32], *psych* [27], and *MASS* [33] and used the full dataset (Final_COVIDiSTRESS_Vol2_cleaned.csv). Based on Lieberoth et al. and Han [15, 19], which conducted similar cross-country multilevel modelling, only responses from language groups with greater than 100 participants were included during measurement invariance testing and alignment. First, we tested for measurement invariance of the Compliance Scale with multi-group CFA (MGCFA). Invariance was tested by comparing fit indicators (RMSEA, SRMR, CFI, TLI). As in previous research [25], our predefined indicator changes were as follows: for metric invariance, less than −0.01 CFI, +0.015 RMSEA, and + 0.030 SRMR; for scalar invariance, less than −0.01 CFI, +0.015 RMSEA, and + 0.015 SRMR [24].

#### Alignment

If measurement invariance was not found, we applied multi-group alignment to adjust factor loadings, intercepts, and group means across groups to resolve variance across languages. We employed *sirt* package in R to perform measurement alignment [32]. If *R^2^* values exceeded 75%, we assumed the achievement of scalar invariance through measurement alignment [25, 34]. Once the alignment was done, we tested whether the alignment process was successfully completed through simulations. To test this alignment, we ran simulations with *n* = 100, 200, and 500 per group across 500 replications and used indicators employed by Han et al. [20]. First, we calculated the aforementioned *R^2^* values for loadings and intercepts across simulations. Second, we examined the correlation between factor means, *cor* (mean), estimated by MGCFA and those by alignment. In the same manner, the correlation between factor variances, *cor* (var), from MGCFA and those from alignment were also examined.

## Results

### EFA of English survey

The best solution was achieved after the stepwise removal of the two pre-determined items. These items included one reverse-scored item (item 7) that Consortium members had previously determined might be difficult for participants to interpret (i.e., ‘Met with people outside of your household for non-essential reasons’) and one item which asked about outdoor mask use (item 3; i.e., ‘Wore a face covering in public when outdoors (e.g., in the street or park)’), a guideline that varied across regions. Because both items were thought to reflect differences in local guidelines, analysis was performed with and without them.

First, we conducted a seven-item EFA (item 7 excluded). The data was suitable for factor analysis. Bartlett’s test of sphericity was significant at an alpha level of 0.05, 𝜒^2^(21) = 1,655.23, *P* < 0.001, indicating that the correlation structure is adequate for factor analyses. The overall Kaiser-Meyer-Oklin (KMO) value verified the sampling adequacy for the analysis, KMO = 0.842. A single-factor solution was most plausible using our criteria, the Hull method with CAF [35], and Kaiser’s criterion of eigenvalues greater than 1 [36], so we ran a Principal Axis Factoring with no rotation. All factor loadings were greater than 0.4 [37, 38]. The model fit was acceptable, CAF(14) = 0.49, reliable (Cronbach’s ɑ = 0.81), and the items accounted for 39.8% of the variance. As indicated by other indices (indicated in our R code), other possible factor solutions could be a 1- or 3-factor model.

We also conducted a six-item EFA (items 3 and 7 excluded). The data was suitable for factor analysis. The Bartlett’s test of sphericity was significant at an alpha level of 0.05, 𝜒^2^(15) = 1,304.82, *P* < 0.001, and the overall KMO value was meritorious, KMO = 0.825. Using the same criteria as above, a single-factor solution was most plausible so we ran a Principal Axis Factoring with no rotation. All factor loadings were greater than 0.4. The model fit was acceptable, CAF(9) = 0.45, and the items accounted for 40.4% of the variance. The results of both EFA analyses are presented in Table 1.

**Table 1.** Principle axis factoring solutions for 7 and 6 Item EFA of English survey

Item	Item #	Factor Loadings for 7-Item Solution	Factor Loadings for 6-Item Solution
Washed your hands regularly	1	0.486	0.495
Wore a face covering in public when indoors (e.g., in a supermarket or cafe)	2	0.487	0.472
Wore a face covering in public when outdoors (e.g., in the street or park)	3	0.604	–
Stayed the recommended distance (for example 2 metres/6 feet) from people who are not part of your household	4	0.788	0.786
Stayed at home unless going out for essential reasons (e.g., buying essentials, doing essential work, or exercising)	5	0.740	0.706
Self-isolated (quarantine) if you suspected that you had been in contact with the virus	6	0.562	0.601
Stayed away from crowded places generally	8	0.682	0.690

*Note:* *N* = 795. Extraction method was principal axis factoring with no rotation. All factor loadings were greater than 0.4.

Because we had pre-determined items 3 and 7 to be less aligned theoretically with globally consistent compliance guidelines, we determined that the six-item solution was the strongest scale and we proceeded with that version. The final solution resulted in one factor with an eigenvalue greater than 1, and all remaining items loaded onto that factor. Internal consistency on the English survey with this sample was adequate (ɑ = 0.79).

### CFA of English survey

CFA was performed on the six-item Compliance Scale in English with the remaining half of the English-speaking sample, and the factor structure was confirmed. The single-factor solution found with EFA was confirmed, scaled CFI = 0.974, scaled RMSEA = 0.045, SRMR = 0.030, scaled TLI = 0.956. All factor loadings were significant, *P* < 0.001. All resulting standardised factor loadings, which reflect the correlations between each indicator variable and the latent factor, for the six items are presented in Table 2.

**Table 2.** Confirmatory factor analysis of English survey

Item	Item #	Standardised Factor Loadings CFA	M(SD)
Washed your hands regularly	1	0.607	6.2(1.1)
Wore a face covering in public when indoors (e.g., in a supermarket or cafe)	2	0.701	6.5(1.1)
Stayed the recommended distance (for example 2 metres/6 feet) from people who are not part of your household	4	0.810	5.4(1.7)
Stayed at home unless going out for essential reasons (e.g., buying essentials, doing essential work, or exercising)	5	0.771	5.2(2.0)
Self-isolated (quarantine) if you suspected that you had been in contact with the virus	6	0.723	5.9(1.6)
Stayed away from crowded places generally	8	0.745	5.8(1.6)

*Note:* *N* = 795. All factor loadings were greater than 0.4.

### Invariance testing and alignment

CFA was repeated with the entire sample across all languages (*N* = 15,740, 15,103 after retaining those with languages ≥100). Invariance testing revealed good fit indices for the configural model, scaled CFI = 0.942, scaled RMSEA = 0.074, SRMR = 0.049, scaled TLI = 0.904. However, when metric invariance was tested, fit indicator changes revealed that metric invariance could not be achieved, scaled CFI = 0.887, scaled RMSEA = 0.083, SRMR = 0.083, scaled TLI = 0.877, Δ scaled CFA = −0.055, Δ scaled RMSEA = +0.09, Δ SRMR = +0.034, Δ scaled TLI = −0.027. Therefore, measurement alignment was conducted. Measurement alignment and simulation results indicated that the alignment of the Compliance Scale was good with *R^2^_loadings_* = 0.965 and *R^2^_intercepts_* = 0.9996.

### Testing alignment

We repeated simulations to examine the alignment in R (see Han et al. [20] for methodological further details). To test this alignment, we ran simulations with *n* = 100, 200, and 500 per group across 500 replications. Using indicators proposed by the original inventors of alignment, Muthén and Asparouhov [39], and employed by Lieberoth et al. [15], we found that alignment of the six-item Compliance Scale worked well with this method. Indicators are presented in Table 3 and demonstrate

1. correlation between latent means estimated by MGCFA with equal loading and intercept constraints and those estimated by alignment
2. correlation between latent variances estimated by MGCFA with equal loading and intercept constraints and those estimated by alignment
3. *R^2^_loadings_*
4. *R^2^_intercepts_*

**Table 3.** Alignment simulation indicators for the Compliance Scale

	*n* = 100		*n* = 200		*n* = 500	
	*M*	*SD*	*M*	*SD*	*M*	*SD*
*cor* (mean)	0.98	0.01	0.98	0.01	0.98	0.01
*cor* (var)	0.93	0.03	0.94	0.03	0.94	0.02
*R^2^_loadings_*	0.95	0.02	0.96	0.01	0.96	0.00
*R^2^_intercepts_*	0.98	0.00	0.98	0.00	0.98	0.00

*Note: cor* (mean) = correlation between latent means estimated by MGCFA with equal loading and intercept constraints and those estimated by alignment; *cor* (var) = correlation between latent variances estimated by MGCFA with equal loading and intercept constraints and those estimated by alignment.

All correlation coefficient values and *R^2^* values exceeded 0.90 in all cases. In particular, cor (mean), which was employed as a core test indicator by Han et al. [20], was higher than 0.95 as required by Lieberoth et al. [15]. *R^2^* values were also higher than 0.75 as well. These simulation results suggest that alignment was properly conducted, and non-invariance was successfully addressed.

### Convergent validation of the Compliance Scale

Convergent validation of the Compliance Scale was conducted by testing the correlation between the six-item scale and vaccine willingness, controlling for vaccine attitudes. The two variables were found to be correlated, *R* = 0.33, *P* < 0.001.

### Known-groups validation of the Compliance Scale

Two known-groups analyses were conducted to test the validity of the Compliance Scale using age and gender t-test comparisons. As predicted, women were found to be more compliant than men, *t*(6,545.7) = −7.79, *P* < 0.001, Cohen’s d = −.16, log(BF) = Infinite. Elderly adults were found to be more compliant than young adults, *t*(962.57) = −7.93, *P* < 0.001, Cohen’s d = −0.30, log(BF) = Infinite. A positive correlation between all ages and compliance scores was found to be significant, *r* = 0.13, *P* < 0.001, log(BF) = 95.05.

## Discussion

We found evidence that the six-item Compliance Scale from the COVIDiSTRESS II Global Survey is reliable and valid. We tested the validity of the Compliance Scale using EFA, CFA, measurement invariance testing and alignment across languages, and convergence with vaccine willingness.

### English version of the Compliance Scale

Exploratory factor analysis of the English version of the scale revealed that the six-item version of the scale is preferable to the full scale and reflects one underlying latent variable. The internal consistency was adequate and factor loadings of each item were significant, which indicates that the items on the scale were reflecting one construct.

Our six-item compliance survey combines the items adapted from previous scales and guidelines that were most relevant and related to international guidelines to reduce the spread of COVID-19 [2]. It builds on and expands previous validation studies of global compliance scales [15, 17] by enhancing content validity prior to data collection and validating across multiple languages.

### Measurement Invariance and novel alignment testing of the cross-language scale

We conducted a measurement invariance test to determine whether the different language versions of the scale are measuring the same construct with the same measurement structure. Invariance testing indicated that measurement alignment was needed to ensure that the scales are comparable across languages and cultures. After alignment, we employed a novel R code to run a Monte Carlo simulation for alignment validation. We used indicators employed by Lieberoth et al. [15]. We found that the alignment of the six-item Compliance Scale worked well with this method. This method can be conducted freely in R and employed for future cross-language surveys.

### Content validity of the scale

Although designing a scale that encompasses multiple compliance behaviours that are fairly consistent across countries is difficult, we have attempted to overcome the challenges posed by previous studies. While previous compliance scales have either involved general questions to allow for generalisations across countries (e.g., ‘I have done everything I could possibly do as an individual to reduce the spread of Coronavirus’; 22) or focused a narrow set of behaviours related to one or two aspects of compliance, our goal was to design a survey that included specific behaviours that captured compliance across the majority of global health guidelines.

To accomplish this, global health guidelines were consulted [2] along with a literature review of previous compliance scales for both COVID-19 and other epidemics (e.g., [40, 41]). Additionally, the COVIDiSTRESS Global Consortium, a group of over 150 researchers representing over 50 countries, met to discuss and review these questions to ensure that the guidelines applied to most countries at the time of survey administration. Questions were modified to be more generalisable; for instance, distance was modified to include both the concurrent WHO guidelines in meters and in feet [2].

Two of the items on the Compliance Scale were known to the researchers to be problematic due to reverse scoring and regional differences in guidelines: meeting with people for non-essential reasons and wearing face coverings outdoors. These questions were included on the original scale because they were of interest for planned analyses at the time of survey administration, but these two guidelines were changing in some regions prior to this analysis. The first was completely excluded from the analysis due to difficulties in interpreting the wording with reverse scoring and because essential reasons for meeting people might be interpreted differently across regions. The second was evaluated with EFA first by inclusion, then by pre-determined exclusion in the subsequent analysis. While face coverings were a well-known preventative measure in most areas, recommendations for wearing masks outdoors varied over regions and over time [42]. Consistent with our prediction, the exclusion of this item proved to be the best solution.

Although the WHO guidelines have changed slightly throughout the COVID-19 pandemic, certain themes have remained consistent both over time and regarding other diseases. Even 3 years into the pandemic, these behaviours are still listed on the WHO guidelines: keeping a physical distance even from others who do not appear to be sick; avoiding crowds and close contact; wearing masks, especially in poorly ventilated environments (e.g., indoors); washing hands frequently with alcohol or soap and water; staying home and self-isolating after showing symptoms or testing positive for COVID-19 [43]. Thus, the items remaining on the short version of our validated survey reflect both the guidelines the WHO had administered at the time and those which have largely remained in effect during local outbreaks. As such, this survey applies to both reducing the spread of COVID-19 and to future viral infections, a benefit over previous health compliance scales.

Our assumption that these items reflect a single latent variable and can be measured on one scale was confirmed with CFA. All of the items grouped together during the factor analysis with significant loadings, demonstrating that they reflect a single underlying variable. Thus, this survey includes specific behaviours that have been consistently recommended by the WHO over time, that generalise across countries and to future health crises, and that pattern together to reflect a single underlying construct of compliance with health guidelines.

### Convergent validity of the scale

Once the scale was found to be reliable and aligned across languages, we then assessed the convergent validity of the scale. A significant correlation between compliance and vaccine willingness was expected, but was not predicted to be close to 1, as these are related but distinct concepts. As predicted, COVID-19 compliance, as measured by the six-item scale, correlated with vaccine willingness.

### Known-groups validity of the scale

Both age and gender have robust, replicated associations with compliance (i.e., higher compliance for elderly adults than younger adults and higher compliance for women than men; [6, 7]). Therefore, to test the validity of the survey, we compared compliance scores in men versus women and in young adults versus elderly adults. As expected, higher compliance scores were found in women and elderly adults than in men and young adults, respectively. To be thorough, we also conducted a correlation analysis between age and compliance scores and found the predicted direction of effects. Thus, all validity measures we conducted support the validity of the scale.

### Limitations

It should be noted that one limitation of the study was the fact that we used snowball sampling methods that may have contributed to a biased sample. This is especially the case as participation was uncompensated and voluntary and may therefore have resulted in a self-selected sample with high compliance. We therefore suggest that overall compliance values in this dataset be interpreted with caution. In addition, we should note that although we showed reliability in translated versions and while the survey translators worked to address language-specific grammar and adapt cultural concepts, we do not yet know whether these translations conveyed the same concepts as the English version intended. A future translatability assessment is advised (e.g., [44]). In addition, local health guidelines differ, so it would be nearly impossible to create a scale with globally consistent compliance guidelines. To mitigate this, a group of 150 researchers from over 50 countries debated and agreed on the items before the survey was administered. Items were also adapted from previous compliance scales and global guidelines by the WHO [2] that have remained largely consistent over time [43]. Finally, validity testing was conducted with another self-report measure, vaccine willingness, which was self-reported and collected as a single item, but further validity testing with additional known-groups measures further strengthened support for the validity of this scale.

## Conclusion

In conclusion, we tested the reliability and validity of the six-item Compliance Scale. This scale measures compliance with health guidelines to reduce the spread of COVID-19. After alignment, the scale was found to be reliable in the full dataset collected across 115 countries and 28 languages (with *N* ≥ 100). Validity testing of the scale was also successful, as the predicted correlation between compliance and vaccine willingness was found and known groups scored as expected on the scale. Therefore, this scale can be used in large-scale studies of compliance across cultures, languages, and nations, providing a valuable tool for global studies and international comparisons.

Not only did we test the validity and reliability of the Compliance Scale, but we also developed a novel R code to conduct Monte Carlo simulations to test alignment. This method allows for alignment testing in R that is comparable to that achieved with other platforms such as MPlus.

## Data Availability

The data and code that support the findings of this study are openly available in GitHub at https://github.com/Neuropinklab/Identity-Project.

## Supplementary material

For supplementary material accompanying this paper visit http://doi.org/10.1017/S0950268823001103. click here to view supplementary material
