# Supplementary material for: Cross-language validation of COVID-19 Compliance Scale in 28 languages
Source: Epidemiol Infect. 2023 Jul 10;151:e119. doi: 10.1017/S0950268823001103 (PMC10468815; doi:10.1017/S0950268823001103)
Supplement: Supplementary file 1 [file S0950268823001103sup001.docx]

Supplementary Materials

Supplementary Table 1

*Compliance Surveys*

| **Author** | **Name of scale** | **Language of scale** | **Number of Items in the scale** | **Scale measuring** |
| --- | --- | --- | --- | --- |
| Banai, Banai, & Mikloušić (2021) | Compliance with official COVID-19 guidelines scale | Available in English (administered in Croatia) | 8 | COVID-19 guidelines |
| Asnakew, Asrese, & Andualem (2020) | Compliance with prevention measures | Unknown (administered in Ethiopia) | 11 | Preventive measures (hygiene and personal protective equipment, social distancing, and travel avoidance) |
| Rubin et al. (2014) | Influenza Telephone Survey Template (FluTEST) | English (administered in Great Britain) | Multiple scales | Avoidance, cleaning, hand-washing behaviours related to flu transmission |
| Vignoles et al. (2021) | Protective and Prosocial Actions | English | Multiple scales | Protective behaviors (personal hygiene, physical distancing) and prosocial actions (helping proximal and distal others) |
| Gustavsson, & Beckman (2020) | Recommendations about Pandemic | Unknown (administered in Sweden) | 8 | COVID-19 recommendations |
| Plohl & Musil (2020) | Compliance with COVID-19 prevention guidelines scale | English/Unknown (administered globally) | 11 | COVID-19 prevention guidelines |
| Toussaint et al. (2020) | Clean and Contain Scale | English | 9 | COVID-19 spread prevention |
| van Bavel et al. (2022) | Physical contact (and spatial distancing) | English available (administered in 67 countries and territories) | 5 | Physical contact |
| van Bavel et al. (2022) | Physical hygiene | English available (administered in 67 countries and territories) | 5 | Physical hygiene |
| Lin et al. (2021) | Self-reported compliance with public health measures | English and translations (dataset from van Bavel et al, 2022) | 10 | See also van Bavel (2022) for combination of physical hygiene and spatial distancing |
| Xu & Cheng (2021) | Mask-wearing behavior and attitude scale | English (United States) | 3 | Mask mandate |
| Xu & Cheng (2021) | Social distancing compliance scale | English (United States) | 5 | Social distancing |
| Xie et al. (2020) | Social distancing compliance scale | English (United States) | 9 | Costs and benefits of social distancing |
| Tung & Thanh (2020) | Compliance with public health safety measure | Unknown (administered in Vietnam) | 9 | Public health safety |
| Wang et al. (2021) | Acceptance of vaccination/behaviours | Unknown (administered in Hong Kong) | 4 | Vaccination and compliance |
| Yamada et al. (2021) | Compliance | English and 46 translations (administered in 176 countries) | 6 | Compliance with local prevention guidelines |
| Clark, Davila, Regis, & Kraus (2020) | Rule following scale | English, French, Italian, Portuguese, Mandarin, Spanish, German (administered internationally) | 3 | Self-isolation |
| Clark, Davila, Regis, & Kraus (2020) | Health precautions scale | English, French, Italian, Portuguese, Mandarin, Spanish, German (administered internationally) | 4 | Public health precaution |
| Clark, Davila, Regis, & Kraus (2020) | Giving health advice scale | English, French, Italian, Portuguese, Mandarin, Spanish, German (administered internationally) | 5 | Spreading awareness |
| This Dataset: Blackburn et al. (2022) | Compliance Scale | English and 48 translations, (administered globally in 137 countries) | 8 | Compliance with COVID-19 WHO guidelines |

**References**

Asnakew, Z., Asrese, K., & Andualem, M. (2020). Community risk perception and compliance with preventive measures for COVID-19 pandemic in Ethiopia. *Risk Management and Healthcare Policy, 13*, 2887–2897. <https://doi.org/10.2147/RMHP.S279907>

Banai, I. P., Banai, B., & Mikloušić, I. (2021). Beliefs in COVID-19 conspiracy theories, compliance with the preventive measures, and trust in government medical officials. *Current Psychology*, 1–11. <https://doi.org/10.1007/s12144-021-01898-y>

Blackburn, A.M., Vestergren, S. & the COVIDiSTRESS II Consortium. (2022). COVIDiSTRESS diverse dataset on psychological and behavioural outcomes one year into the COVID-19 pandemic. *Sci Data*, *9*, 331. <https://doi.org/10.1038/s41597-022-01383-6>

Clark, C., Davila, A., Regis, M., Kraus, S. (2020). Predictors of COVID-19 voluntary compliance behaviours: An international investigation. *Global Transitions, 2*, 76-82. <https://doi.org/10.1016/j.glt.2020.06.003>

Gustavsson, J., & Beckman, L. (2020). Compliance to recommendations and mental health consequences among elderly in Sweden during the initial phase of the COVID-19 pandemic: A cross sectional online survey. *International Journal of Environmental Research and Public Health, 17*(15), 5380. <https://doi.org/10.3390/ijerph17155380>

Lin, T., Harris, E. A., Heemskerk, A., Bavel, J. J. van, & Ebner, N. C. (2021). A multi-national test on self-reported compliance with COVID-19 public health measures: The role of individual age and gender demographics and countries’ developmental status. *Social Science & Medicine, 286,* 114335. <https://doi.org/https://doi.org/10.1016/j.socscimed.2021.114335>

Plohl, N. & Musil, B. (2020). Modeling compliance with COVID-19 prevention guidelines: The critical role of trust in science, *PsyArcXiv 26*(1), 1. <https://doi.org/10.31234/OSF.IO/6A2CX>

Rubin, J., Bakhshi, S., Amlôt, R., Fear, N., Potts, H., & Michie, S. (2014). The design of a survey questionnaire to measure perceptions and behaviour during an influenza pandemic: the Flu Telephone Survey Template (FluTEST). *Health Services and Delivery Research, 24*. <https://www.ncbi.nlm.nih.gov/books/NBK263566/>

Toussaint, L. L., Cheadle, A. D., Fox, J., & Williams, D. R. (2020). Clean and Contain: Initial Development of a Measure of Infection Prevention Behaviors During the COVID-19 Pandemic. *Annals of Behavioral Medicine, 54*(9), 619–625. <https://doi.org/10.1093/abm/kaaa064>

Tung, L. T., & Thanh, P. T. (2020). Survey data on government risk communication and citizen compliance during the COVID-19 pandemic in Vietnam. *Data in Brief, 33,* 106348. <https://doi.org/10.1016/J.DIB.2020.106348>

van Bavel, J. J., Cichocka, A., Capraro, V., Sjåstad, H., Nezlek, J. B., Pavlović, T., Alfano, M., Gelfand, M. J., Azevedo, F., Birtel, M. D., Cislak, A., Lockwood, P. L., Ross, R. M., Abts, K., Agadullina, E., Aruta, J. J. B., Besharati, S. N., Bor, A., Choma, B. L., … Boggio, P. S. (2022). National identity predicts public health support during a global pandemic. *Nature Communications, 13*(1), 1–14. <https://doi.org/10.1038/s41467-021-27668-9>

Vignoles, V., Jaser, Z., Taylor, F., & Ntontis, E. (2021). Harnessing shared identities to mobilize resilient responses to the COVID-19 pandemic. *Political Psychology*, Early view. <https://www.doi.org/10.1111/pops.12726>

Wang, K., Wong, E. L. Y., Ho, K. F., Cheung, A. W. L., Yau, P. S. Y., Dong, D., Wong, S. Y. S., & Yeoh, E. K. (2021). Change of Willingness to Accept COVID-19 Vaccine and Reasons of Vaccine Hesitancy of Working People at Different Waves of Local Epidemic in Hong Kong, China: Repeated Cross-Sectional Surveys. *Vaccines, 9*(1), 62. <https://doi.org/10.3390/VACCINES9010062>

Xie, Weizhen, Campbell, Stephen & Zhang, Weiwei. (2020). Working memory capacity predicts individual differences in social-distancing compliance during the COVID-19 pandemic in the United States. *PNAS, 117*(30), 17667-17674. <https://doi.org/10.1073/pnas.2008868117>

Xu, Ping & Cheng, Jiuqing (2021) Individual differences in social distancing and mask-wearing in the pandemic of COVID-19: The role of need for cognition, self-control and risk attitude, *Personality and Individual Differences*, 175. <https://doi.org/10.1016/j.paid.2021.110706>

Yamada, Y. et al. (2021). COVIDiSTRESS Global Survey dataset on psychological and behavioural consequences of the COVID-19 outbreak. *Scientific Data,* *8,* 1-23, <https://doi.org/10.1038/s41597-020-00784-9>
